# Analysis of Tuberculosis Meningitis Pathogenesis, Diagnosis, and Treatment

**DOI:** 10.3390/jcm9092962

**Published:** 2020-09-14

**Authors:** Aysha Arshad, Sujay Dayal, Raj Gadhe, Ajinkya Mawley, Kevin Shin, Daniel Tellez, Phong Phan, Vishwanath Venketaraman

**Affiliations:** College of Osteopathic Medicine of the Pacific, Western University of Health Sciences, Pomona, CA 91766-1854, USA; aysha.arshad@westernu.edu (A.A.); sujay.dayal@westernu.edu (S.D.); raj.gadhe@westernu.edu (R.G.); ajinkya.mawley@westernu.edu (A.M.); seungkevin.shin@westernu.edu (K.S.); daniel.tellez@westernu.edu (D.T.); phong.phan@westernu.edu (P.P.)

**Keywords:** tuberculosis meningitis, meta-analysis, TBM, glutathione, Western University of Health Sciences

## Abstract

Tuberculosis (TB) is the most prevalent infectious disease in the world. In recent years there has been a significant increase in the incidence of TB due to the emergence of multidrug resistant strains of *Mycobacterium tuberculosis* (*M. tuberculosis*) and the increased numbers of highly susceptible immuno-compromised individuals. Central nervous system TB, includes TB meningitis (TBM-the most common presentation), intracranial tuberculomas, and spinal tuberculous arachnoiditis. Individuals with TBM have an initial phase of malaise, headache, fever, or personality change, followed by protracted headache, stroke, meningismus, vomiting, confusion, and focal neurologic findings in two to three weeks. If untreated, mental status deteriorates into stupor or coma. Delay in the treatment of TBM results in, either death or substantial neurological morbidity. This review provides latest developments in the biomedical research on TB meningitis mainly in the areas of host immune responses, pathogenesis, diagnosis, and treatment of this disease.

## 1. Introduction

Worldwide, Tuberculosis (TB) remains the most important infectious disease in causing morbidity and death [1]. About one-third of the population worldwide has currently contracted TB infection through *Mycobacterium tuberculosis* [1]. Recently, the WHO reported that there are about eight million new TB cases yearly. In addition, the incident of TB is expected to increase [1]. Notably, patients who suffer from immunosuppression are much more likely to contract extra-pulmonary tuberculosis [2,3,4], and of the extra pulmonary variants of TB, TBM shows the highest mortality rate [5,6,7,8].

TBM is characterized as a severe manifestation of TB and usually requires emergent intervention, due to the quick hematogenous dissemination of the tuberculosis bacillus. This dissemination is quickly advanced and seen clinically with focal neurological defects, altered mental status, cerebral infarcts, prolonged fever, and highly likelihood of stroke. The presence of stroke is a general indicator of basal ganglia damage and a poor prognosis at three months [3,4,5,6,7,8]. Unfortunately, TBM is difficult to diagnose due to its clinical similarity with other neurological disease manifestations. It has been concluded in several research articles that a better prognosis is expected through early treatment and diagnosis. Failure to do so is highly associated with death or severe neurological impairment [3,4,5,6,7,8].

## 2. Methodology

TBM is considered the most severe form of TB. It is our intention to better understand the host-pathogen interactions, pathogenesis, diagnostics, and treatment modalities to bridge the knowledge gap between different fields and better understand TBM as a clinical threat to humanity. To do this, our group of 8 co-authors decided to conduct an extensive PubMed search to analyze 77 articles relating to different aspects of TBM. These articles were specifically selected by relevance of the topic and credibility of the source (Table 1). While, the majority of the information is from past research and literature, we tried introducing novel concepts as can be exemplified in Section 4 adjunctive therapy. Keywords that were used to obtain these articles were as follows: TBM, extrapulmonary TB, cerebrospinal TB, HIV and TB, clinical diagnosis of TBM, laboratory diagnosis of TBM, current treatments of TBM and potential treatments of TBM. We decided to exclude the following in our searches: Bacterial meningitis, viral meningitis, fungal meningitis, TBI. To allow us to keep the information on TBM as recent as possible, we selected articles with a publish date no later than 1970 (Table 1). Finally, to maintain fluidity and uniformity within the project, any disputes that arose were solved by discussion amongst the group members initially, with a final decision from Dr. Venketaraman.

## 3. Results

### 3.1. Host-Immune Responses against Infection

*M. tuberculosis* infection is usually acquired by inhalation of infectious aerosol particles containing the pathogen [9]. Most individuals in the general population who become infected with *M. tuberculosis* do not develop clinical disease (active pulmonary TB), due to the concerted effector mechanisms mounted by the cells of innate and adaptive immune systems, which results in *M. tuberculosis* becoming dormant. This condition is commonly referred to as latent tuberculosis infection (LTBI) [10]. Little is known about what happens during the early phase of immunity against *M. tuberculosis* infection, even before the pathogen is encountered by the phagocytic cells. Myeloid dendritic cells and macrophages are considered to provide initial first-line defense against *M. tuberculosis* infection. Once dendritic or alveolar macrophages encounter *M. tuberculosis*, the bacteria are recognized via microbe-associated molecular patterns (MAMPs) by toll-like receptors (TLRs) on the host phagocytic cells. The interaction between MAMPs and TLRs trigger cell signal transduction that induces a proinflammatory response. However, *M. tuberculosis* has evolved mechanisms to subvert these host responses for its own survival in the host [11,12].

TLR2 and TLR4 on the host phagocytic cells are important for recognizing *M. tuberculosis* MAMPs. *M. tuberculosis* growth can be inhibited in both mouse and human macrophages by activation of the TLR2 [12]. However, the inhibition in the growth of *M. tuberculosis* in the mouse macrophage was dependent on the intracellular nitric oxide pathway, whereas in the human macrophage, it was independent on the nitric oxide pathway [13].

Further characterization of the mechanism of killing of *M. tuberculosis* in human macrophages has shown that TLR2 activation up-regulates the expression of the vitamin D receptors as well as vitamin D-1 hydroxylase. The expression of vitamin D receptor-related genes leads to an increased expression of cathelicidin, an antimicrobial peptide, which is then responsible for inhibiting the growth of *M. tuberculosis* [14,15].

TNF-α seems to be involved in formation and maintenance of granuloma. In conjunction with IFN-γ, TNF-α can enhance the effector responses against *M. tuberculosis* infection [16].

### 3.2. Pathogenesis of TB Meningitis

Extrapulmonary TB begins when the bacteria disseminate from the lungs to the lymph nodes, and during this time, there is bacteremia which seed *M. tuberculosis* to other organs in the body for TB, specifically the meninges and the brain parenchyma [17,18]. During hematogenous dissemination, mycobacteria may be deposited adjacent to the ventricles or subarachnoid space, leading to granuloma formation at those sites of deposition [19,20]. *M. tuberculosis* can breach the blood brain barrier (BBB) extracellularly or intracellularly via dendritic cells or macrophages [17,21].

TBM occurs when subependymal or subpial tubercles, also known as “rich foci” seed during bacillemia of primary infection or disseminated disease [17,18,22,23]. This rupture of the granuloma into the subarachnoid space leads to an intense inflammatory response, which eventually causes meningitis [18,19,21,23]. The tissue damage seen in the brain is due to a host inflammatory response rather than over-replication in the CSF [21,23].

The inflammatory response is due to rupture which includes a collection of a tuberculous, thick gelatinous exudate (erythrocytes, mononuclear cells, neutrophils, and bacilli) at the basal brain and vasculitis within the cerebral arterial system, including branches of the middle cerebral artery, the vertebrobasilar system, and the vessels of the Circle of Willis [18,19,24]. All of this can lead to long-term neurological defects from either infarction or compression by the exudate, which can encase cranial nerves and cause nerve palsies, entrapment of blood vessels, and blocking of CSF flow in the cerebral aqueduct to cause hydrocephalus. These processes produce adhesions, obliterative vasculitis (internal carotid, middle cerebral arteries), and encephalitis [18,19,23].

On average, TBM occurs 6 to 12 months after the primary infection, and patients show a prolonged inflammatory response [24]. The risk factors for TBM include malignancy, malnutrition, alcoholism, HIV, the use of immunosuppressive agents, and cortisol deficiency [17,25].

TBM can eventually lead to intracranial tuberculomas in an immunocompromised patient [17,26]. Focal neurological signs result from formation of tuberculomas and abscess after infection, with basal ganglia the most common site of infarction [26].

Although the role of TNF--α is crucial for the formation of granuloma and enhanced killing of infected cells in the lungs during the primary infection, concentrations of TNF-α in CSF correlate with clinical correlation of TBM [16,18]. Intervention with thalidomide, an anti-TNF agent, resulted in an improvement in survival and neurological outcome due to TBM [18].

One study showed that there was also significant elevation of cathelicidin LL-37, interleukin (IL)-13 and vascular endothelial growth factor (VEGF) and reduction of IL-17 in the CSF of children with TBM, compared to children with viral and bacterial meningitis [36]. This biomarker pattern suggests a host immune response which is disease-specific and may be of diagnostic and therapeutic importance [24].

### 3.3. Clinical Presentation of TB Meningitis

The clinical presentations of TBM have many similar features to those of generalized bacterial meningitis, which include, but are not limited to, headache, fever, stiff neck, nausea, and vomiting. However, according to data obtained from many clinical trials, there are clinical features that are present more commonly in TBM than in generalized bacterial meningitis and may have values in distinguishing TBM in clinical practice. The presence of neurologic signs and symptoms are frequently observed. In a study involving 160 patients, the frequency of altered mental status, change in personality, and coma were noted in 59, 28, and 21 percent of patients, respectively [27]. Cranial nerve palsy is also common and most frequently involve cranial nerve II, which affects vision, and cranial nerve VI, which affects lateral movement of the eyeball. The frequency of cranial nerve palsy was observed in 33 percent of patients in a studying involving 158 patients [28].

TBM has three clinically distinct phases, which are the prodromal phase, the meningitic phase, and the paralytic phase. In the prodromal phase, which lasts from one to three weeks, patients experience nonspecific signs and symptoms, which include, but are not limited to, malaise, headache, low-grade fever, and change in personality. In the meningitic phase, patients experience more prominent neurologic signs, which include nausea, vomiting, headache, lethargy, confusion, and cranial nerve palsies. Finally, in the paralytic phase, the illness progresses quickly, and patients can deteriorate into coma, seizure, and possibly paralysis. For patients in this stage, death follows quickly if they are not treated [29,30,31,32].

Aside from the typical presentations above, TBM can also present atypically in some patients, potentially mimicking other neurologic conditions, complicating the diagnosis and treatment. Instead of an acute condition, it can present as a slowly progressing dementia over a period of years, potentially mimicking Alzheimer, and characterized by personality change, social withdrawal, memory deficits, and impaired executive functions. Alternatively, patients can present with signs of encephalitis instead of meningitis. Signs and symptoms of encephalitis include coma and convulsions [33].

TBM can appear in patients who have no previous history of signs and symptoms from *M. tuberculosis* infection. In one paper studying 61 patients with TBM, only 6 patients reported that they were aware of previous *M. tuberculosis* infection [34].

### 3.4. Diagnosis

The conundrum of TBM arises due to the need for rapid diagnosis for better outcomes. However, it is difficult to do so. We will analyze the following diagnosis paradigms: CSF content, acid-fast smear, lumbar puncture, NAAT, and neuroimaging (i.e., MRI) [24].

CSF findings consistent with TBM will reveal leukocytosis, with an increase in protein and decrease in glucose [24]. It is worth noting that the initial presentation will have neutrophil predominance versus chronic etiologies will show lymphocytes [23]. A study illustrated that a CSF glucose concentration of < 2.2 mmol/L had specificity 0.96 and sensitivity 0.68 in diagnosis [35]. That same study also illustrated that protein concentration > 1 g/L had specificity 0.94 sensitivity 0.78 [35].

CSF acid fast smear has shown to have very low sensitivity. However, by performing analysis on several large volume (10–15 mL) samples lumbar punctures daily can increase the sensitivity up to more than 85% [23]. Although, culturing can take several weeks, it should still be carried out to determine the drug sensitivity of the organism [23]. Drug resistant strains have many important prognostic and treatment implications; indeed, TBM due to isoniazid resistant strains have been associated with two time increase in mortality [23].

Nucleic acid simplification testing is the new tool in toolkit for diagnosing TBM [24]. When definite TB meningitis is used as a reference, the sensitivity and specificity for the Xpert MTB/RIF are 39% and 100% in children respectively. A meta-analysis of 14 studies which analyzed the accuracy of NAAT in diagnosing TBM reported a sensitivity of 0.56, specificity of 0.98, negative likelihood of 44, and positive likelihood ratio of 35.1, suggesting their role in confirmation, but not ideal for ruling out TBM [24].

The diagnosis of TBM can be supported by neuroimaging and the class neuroradiologic features of TB meningitis, such as basal meningeal enhancement and hydrocephalus [23]. The incidence of hydrocephalus is higher in children. In a computed tomography study of 60 cases of TBM in both adults and children, 87% of children was reported to have hydrocephalus on imaging, whereas only 12% of adults had hydrocephalus [18]. The CT showed infarcts in 28%, 83% of which occurring in the middle cerebral artery territory [36]. All patients showed signs of basal enhancement [24]. The MRI findings in HIV-infected children, include high frequency of ventricular dilation following cerebral atrophy, high frequency of communicating hydrocephalus, low frequency of basal meningeal enhancement and granuloma formation [24].

### 3.5. Treatment

The traditional treatment for pulmonary TB has been standardized to the RIPE [rifampin (RIF), isoniazid (INH), pyrazinamide, ethambutol) therapy for 2 months followed by RI (rifampin, isoniazid) for 10 months [20,23,37,38,39]. The empirical treatment for TBM remains the same as the treatment for pulmonary TB; the empirical treatment is warranted when clinical features and CSF findings are suggestive of TBM, even before microbiologic confirmation, since timely treatment dramatically improves the outcome of TBM [20,23].

INH is considered the most critical of the first line agents due to its excellent CSF penetration and high bactericidal activity [23,24]. While, rifampin penetrates the CSF less freely, the high mortality of TBM due to RIF-resistant strains has confirmed its importance [23,24]. Pyrazinamide has excellent penetration into the CSF and is a key drug in reducing the total treatment for drug-susceptible TB. Hence, if pyrazinamide cannot be tolerated, the treatment course for TBM should be lengthened to a total of 18 months [23].

There are many supplements to the RIPE therapy leading to favorable outcomes, most notably fluoroquinolones and corticosteroids. Fluoroquinolones decrease DNA topoisomerase 2 or 4, inhibiting replication and serving as a bactericidal. Corticosteroids are anti-inflammatory and are prominently used for immunosuppression. The newer generation fluoroquinolones, for example, levofloxacin and moxifloxacin, have strong activity against most strains of *M. tuberculosis* and have excellent CSF penetration and safety profiles, thus fluoroquinolones would appear to have great potential as part of first-line therapy for TBM [23]. In a randomized controlled study for TBM treatment, the addition of a fluoroquinolone to a standard regimen enhanced anti-TB performance, as measured by various clinical parameters [23].

Another drug mentioned that decreased the mortality for patients would be thalidomide. Thalidomide decreases TNF-α, a cytokine that activates macrophages to form caseous granulomas in TB. The addition of thalidomide, a potent inhibitor of TNF-α, to antibiotics was superior to antibiotics alone in protecting rabbits from dying (50% reduction in their model of TBM) [4]. Thalidomide is severely teratogenic, resulting in limb defects in the newborn. Consequently, female patients should be screened before the course of thalidomide for precautionary measures. Another agent discussed would be bedaquilline, an agent that inhibits the proton pump of mycobacterial ATP synthase which is responsible for energy generation in *M. tuberculosis*.

It is worth mentioning that patients who present with TB with past medical history conditions are not treated similarly. For example, HIV patients are given the standard regimen of 2 months RIPE followed by 10 months of RI, but also given an ART therapy after [20,23,37,38,39]. Patients who develop syndrome of inappropriate antidiuretic hormone (SIADH) from *M. tuberculosis* infection should engage in fluid restriction and take medications such as furosemide and demeclocycline. A hydrocephalus complication from TB should be treated with surgery, such a shunt to decrease the pressure. Regardless, it is definitive that treating all cases in a standard manner in a continuum can be potentially lethal, thus, a thorough medical history should be obtained prior to starting therapy.

## 4. Discussion

### Adjunctive Therapy

Immuno-adjunctive therapy appears to be promising in improving the outcome of clinical control of refractory mycobacterial infections. Dr. Venketaraman’s research group has reported that individuals with active pulmonary TB exhibit a marked deficiency in glutathione (GSH), the principal non-protein thiol responsible for cellular homeostasis and maintenance of the intracellular redox balance. GSH levels are significantly compromised in peripheral blood mononuclear cells (PBMCs) and red blood cells (RBCs) isolated from individuals with active pulmonary TB and this decrease correlated with increased production of pro-inflammatory cytokines and enhanced growth of *M. tuberculosis* [40]. GSH possesses a direct antimycobacterial activity in vitro and at physiological concentrations (5 mM) [36,41]. In combination with cytokines such as IL-2 and IL-12, GSH enhances the functional activity of natural killer (NK) cells to inhibit the growth of *M. tuberculosis* inside human monocytes [42,43]. Similarly, GSH activates the functions of T lymphocytes to control *M. tuberculosis* infection inside human monocytes [44]. GSH levels have also been shown to be compromised in HIV positive subjects and in individuals with uncontrolled type 2 diabetes (T2DM) who have increased risks for susceptibility to both pulmonary and extrapulmonary TB [42,44,45,46,47,48,49,50]. Importantly, Dr. Venketaraman’s research also demonstrated in the autopsied human brain tissues that the levels of total and reduced forms of GSH were significantly compromised in HIV-1 infected individuals who have increased risks for susceptibility to TBM [51].

Put together, these findings (1) unfold a novel and potentially important innate defense mechanism adopted by human macrophages to control *M. tuberculosis* infection [36,40,41,42,44,45,46,48,49,52] and (2) indicate that GSH controls *M. tuberculosis* infection by functioning as an antimycobacterial agent as well as by enhancing the effector functions of immune cells [36,40,41,42,43,44,45,46,47,48,49,52]. However, the underlying mechanisms by which GSH-deficiency alters the immune responses leading to increased susceptibility to TBM remains unknown, and the potential use of GSH as a possible anti-TB therapeutic agent remains untapped.

## 5. Conclusions

The human host serves as the natural reservoir for *M tuberculosis*. The ability of the organism to efficiently establish latent infection has enabled it to spread to nearly one-third of the world’s population. The underlying mechanisms responsible for successful dissemination of *M tuberculosis* to the meninges to cause TB meningitis remains poorly understood. Given the magnitude of the health problem and the emergence of drug-resistant strains of the organism, a better understanding of the protective immunity and pathogenesis of TB meningitis, development of reliable rapid laboratory diagnosis, therapeutics and effective vaccine are highly desirable.

## Figures and Tables

**Table 1:** Analysis of TB meningitis Pathogenesis, Diagnosis and Treatment.

Article Name	Author	Summary
1. Global TB Report [1].	World Health Organization (WHO)	Report provided information on TB epidemiology, progression in prevention, diagnosis, and treatment.
2. Extrapulmonary tuberculosis in the United States [2].	Rieder	Extrapulmonary TB is largest in children and decreases with increasing age, commonly seen in females and blacks/Asians.
3. Extrapulmonary tuberculosis in patients with human immunodeficiency virus infection [3].	Shafer	HIV infected patients are more likely than control patients to have TB. Although, fever was seen in the patients infected w/TB, the diagnosis was difficult and delayed as there is co-infection w/HIV and other pathologies have to be ruled out. Acid-fast bacteria sputum results were not as accurate (+ in less than 50%). Most immediate diagnostic test is a biopsy.
4. Use of ImiD3, a thalidomide analog, as an adjunct to therapy for experimental tuberculous meningitis. Antimicrobial agents and chemotherapy [4].	Tsenova	Treatment with TB drugs along with IMiD3 limited the changes in patients’ neurology and improved the survival by 73%
5. Presentation and outcome of tuberculous meningitis in a high HIV prevalence setting [5].	Marais	Six month all because mortality is lower in patients who received antiretroviral therapy during their TB treatment course with the hazard ratio at 0.30 (95% CI = 0.08 to 0.82)
6. Oxidative stress and antioxidants in tubercular meningitis [6].	Sudha	The study found that the blood antioxidant levels of TB meningitis patients were low compared to controls, and the levels improved after treatment, suggesting a role of free radicals in TB meningitis
7. Evaluation of free radical status in CSF in childhood meningitis [7].	Ray	Their result suggested that natural and synthetic antioxidants might have a role in preventing disease progression and tissue damage in childhood meningitis
8. Variations of the glutathione level in the cerebrospinal fluid in tuberculous meningitis treated with streptomycin [8].	Mule	TBM is the most common and severe form of extra-pulmonary TB associated with significant mortality. It remains difficult to diagnose due to broad, non-specific clinical spectrum. Clinical features include the following: Cerebral infarct/lesion, fever, headache, neck stiffness, and basal ganglia stroke.
9. Clinical use of anti-TNF therapy and increased risk of infections [9].	Ali	Anti-TNF drugs have emerged as successful agents in the treatment of chronic inflammatory diseases but do possess the risk of opportunistic infections. Patients taking such drugs should be adequately vaccinated and monitored for signs of infection.
10. Role of TNF-Alpha, IFN-Gamma, and IL-10 in the Development of Pulmonary Tuberculosis [10].	Cavalcanti	Cellular immunity such as TH1 cytokines play key role in TB host immune response. TNF alpha and IFN gamma stimulate reactive nitrogen intermediates, mediating tuberculostatic function of macrophages and granuloma formation (disease progression). An increase in IL10 has been implicated with increased survival of mycobacteria.
11. Immunomodulation by vitamin D: implications for TB [11].	Chun	It has been found that Vitamin D deficiency may be linked to increased risk of TB and other immune disorders. This article details cellular and molecular mechanisms of Vitamin D and its potential role in normal and abnormal immune function.
12. Toll-like receptor 2-deficient mice succumb to Mycobacterium tuberculosis infection [12].	Drennan	One of the ways that the immune system combats Mycobacterium infection is by recognition of the immune system. Specifically, in this infection, macrophages use the TLR -2 to eliminate the antigen, it is proposed that mice TLR-2 levels may be associated with mycobacterium infection and improved prognosis in human patients.
13. The Intracellular Environment of Human Macrophages That Produce Nitric Oxide Promotes Growth of Mycobacteria [13].	Jung	NO has a protective role in allowing TB to remain within macrophages via reactive nitrogen intermediates, although the clear mechanism is unknown. Treatment with IFN gamma resulted in increase NOS2/3 isoforms (enzymes that increased NO).
14. Pattern recognition receptors and cytokines in Mycobacterium tuberculosis infection--the double-edged sword [14]?	Hossain	This article studies the paradox of human innate immunity response during M.TB infection. The paradox is explained as Dendritic cells promoting the first step of the immune response by promoting TLRs and PRRs. These receptors and cells through a cascade of biochemical reactions lead to the activiation of IFN-y and TNF-a which regulate inflammation and granuloma formation. It has been noted however that these responses may provide survival to the infecting pathogen.
15. Latent tuberculosis infection: An overview [15].	Kiazyk	Latent TB infection (LTBI) is persistent immune response to TB antigens without evidence of clinical manifestations. People with LTBI can progress to active TB or reactivation, seen increasingly in immunocompromised.
16. The Immune Response in Tuberculosis [16].	O’Garra	CD4 T-cells, IL 12, IFN gamma, and TNF alpha are critical in control on TB infection, but the host factors that determine why some individuals are protected from infection are unclear. Genetic factors may be implicated in increased risk of patients developing active infection.
17. Tuberculous meningitis [17].	Wilkinson	The paper discusses the new understanding about the inflammatory process in TB meningitis
18. Tuberculous meningitis [18].	Thwaites	The paper discusses the current uncertainties with regards to TB meningitis, paying more attention to the diagnosis and treatment
19. Tuberculous Meningitis in Children and Adults: New Insights for an Ancient Foe [19].	Mezochow	The paper states that delayed diagnosis and treatment of TB meningitis can lead to neurologic consequences, such as hydrocephalus, cranial nerve palsy, and seizure
20. Intracerebral Tuberculomas: A Rare Cause of Seizure in an Immunocompetent Young Male [20].	Vu	The paper presents a rare clinical presentation of CNS involvement of TB: intracerebral tuberculoma
21. Pathogenesis of central nervous system tuberculosis [21].	Be	The groups found that unlike other types of bacteria that also cause meningitis, M. tuberculosis do not traverse the blood CSF barrier at the choroid plexus, but instead at the BBB
22. Tuberculous meningitis and miliary tuberculosis: the Rich focus revisited [22].	Donald	The authors suggest that miliary tuberculosis may be directly involved in pathogenesis of TB meningitis since the bacillemia serves to increase the chance that meningeal focus is established, giving rise to TB meningitis
23. Tuberculous Meningitis: Diagnosis and Treatment Overview [23].	Marx	The paper gives overall diagnosis and treatment review of TB meningitis
24. Tuberculous meningitis in children: Clinical management and outcome [24].	Daniel	The paper reports that children that have TB meningitis should be treated with medications against tuberculosis, as well as steroids. They also report that levels of TB medications achieve lower concentration in the CSF of children vs. than in adults.
25. Tuberculous meningitis: advances in diagnosis and treatment [25].	Torok	The paper states that human genetic polymorphism may explain the differences in response to anti-inflammatory therapies
26. Tuberculous meningitis in children is characterized by compartmentalized immune responses and neural excitotoxicity [26].	Rohlwink	The study shows that the disease processes of tuberculosis are different in the peripheral vs. the central nervous system
27. Tuberculous meningitis in adults: a review of 160 cases [27].	Pehlivanoglu	The frequency of altered mental status, change in personality, and coma were noted in 59, 28, and 21 percent of patients, respectively.
28. Incidence, predictors and prognostic value of cranial nerve involvement in patients with tuberculous meningitis: a retrospective evaluation [28].	Sharman	The frequency of cranial nerve palsy was observed in 33 percent of patients in a studying involving 158 patients.
29. Tuberculous meningitis at Cleveland Metropolitan General Hospital 1959 to 1963 [29].	Hinman	The paper states that the discovery of isoniazid in 1952 has decreased the mortality rate of TB meningitis from 100% to between 20% to 50%.
30. Tuberculous meningitis [30].	Kennedy	The paper states that in a study of 52 patients with TB meningitis, 85% recovered, 4% had residual disability, and 15.
31. Tuberculosis of the central nervous system in children: a 20-year survey [31].	Farinha	The paper examined 38 children with CNS tuberculosis and found that overall mortality was 13% and permanent neurological sequelae were seen in 47%.
32. Tuberculous meningitis: a 30-year review [32].	Kent	The paper examined 58 cases of TB meningitis, of which 7% died, 5% developed neurological sequelae.
33. Tuberculous encephalopathy with and without meningitis [33].	Udani	Patients present case in which tuberculosis infection presents as dementia and as encephalitis instead of the classic signs and symptoms of meningitis.
34. Tuberculous meningitis in adults: review of 61 cases [34].	Sutlas	Only 6 patients out of 61 reported that they were aware of previous TB infection.
35. The diagnostic value of cerebrospinal fluid chemistry results in childhood tuberculous meningitis [35].	Solomons	CSF showing elevated protein is well-described in literature as bacterial meningitis, but diagnostic evidence for TBM is lacking. Research done showed a significant decrease in glucose in groups with TBM than groups without. CSF of glucose <2.2 diagnosed TBM w/sensitivity 0.68 specificity 0.96. Research done showed a significant increase in protein in groups with TBM than groups without. CSF of protein >1 diagnosed TBM w/sensitivity 0.78 specificity 0.94.
36. Characterization of a glutathione metabolic mutant of *Mycobacterium tuberculosis* and its resistance to glutathione and nitrosoglutathione [36].	Dayaram	Glutathione is synthesized during production of ROS and NO, and iis directly toxic to M. TB. Study showed that M. TB intracellular survival in macrophages with glutathione mutation.
37. Treatment of Tuberculous Meningitis and Its Complications in Adults [37].	Davis	This article discusses several drugs widely used to treat Tuberculosis Meningitis. It concludes that although many drugs have certain efficacy against the disease, many have not been adequately studied and strong evidence cannot be found as to their ability to cross the blood brain barrier
38. A Case Report on Complicated Tuberculous Meningitis [38].	Jawad	This case follows a 19 years old Asan Female with Tb Meningitis in an attempt to study the importance of early diagnosis and treatment.
39. Tuberculous Meningitis Diagnosis and Treatment in Adults: A Series of 189 Suspected Cases [39].	Luo	This study summarizes the clinical features including imaging diagnostics, treatment and outcomes of Tb Meningitis in a cohort of 189 patients.
40. Glutathione levels and immune responses in tuberculosis patients [40].	Venketaraman	Glutathione levels are significantly reduced in mononuclear cells and RBC isolated from TB patients. Treatment with n-acetyl cystine improves control by decreasing IL 1, IL 6, IL 10, and TNF alpha.
41. Glutathione and nitrosglutathione in macrophage defense against Mycobacterium tuberculosis [41].	Venketaraman	M. TB is sensitive to glutathione, as it controls macrophage replication.
42. Control of Mycobacterium tuberculosis growth by activated natural killer cells [42].	Guerra	Glutathione-enhanced NK cells (activated from n-acetyl cysteine, IL2, and IL 12) inhibit M. TB growth via neutralization of Fas and CD40L.
43. Natural killer cells, glutathione, cytokines and innate immunity against Mycobacterium tuberculosis [43].	Millman	The study found that glutathione in combination with IL-2 and IL-12 improve NK cell functions, helping to control TB infection
44. Adaptive immune responses against *Mycobacterium tuberculosis* infection in healthy and HIV infected individuals [44].	Guerra	T-lymphocytes that are derived from HIV-infected individuals are deficient in glutathione, leading to decreased levels of TH1 cytokines (IL2, IL 12, IFN gamma) and increased growth of M. TB.
45. Glutathione supplementation improves macrophage functions in HIV. Journal of Interferon and cytokine research [45].	Morris	Treatment with n-acetyl cystine or IGSH (glutathione in liposomal formulation) replenishes glutathione and is correlated with a decrease in intracellular growth of M. TB.
46. Unveiling the mechanisms for decreased glutathione in individuals with HIV infection [46].	Morris	Lack of antioxidant activity (oxidized glutathione) and increased free radicals (IL 1, IL 17, TGF beta)/proinflammatory cytokines lead to an increase in M. TB.
47. Glutathione synthesis is compromised in erythrocytes from individuals with HIV [47].	Morris	The study found that levels of enzymes involving in GSH synthesis, such as GSS, GCLC, and GSR were significantly reduced in RBCs isolated from patients with HIV infection, and this correlated with decreased levels of GSH
48. Investigating the causes for decreased levels of glutathione in individuals with type II diabetes [48].	Lagman	The group reports that patients with type 2 diabetes mellitus have lower levels of GSH due to compromised levels of GSH synthesis and metabolism enzymes
49. Liposomal Glutathione Supplementation Restores Appropriate Cytokine Response to Intracellular Mycobacterium tuberculosis Infection in HIV Infected Individuals [49].	Ly	The groups establish a correlation between low level of GSH and increased susceptibility to TB infection, which may be relieved with IGSH supplementation
50. Restoring cytokine balance in HIV Positive Individuals with Low CD4 T Cell Counts [50].	Valdivia	The group found that supplementation with L-GSH in HIV patients whose CD4 + <350 help correct cytokine balance
51. Analysis of Glutathione levels in the Brain tissue samples from HIV-Positive Individuals and subject with Alzheimer’s disease and its implication in the pathophysiology of the disease process [51].	Saing	The group found that the levels of many enzymes involving in the synthesis of GSH were decreased in brain tissue samples from HIV-1 patients
52. Role of glutathione in macrophage control of mycobacteria [52].	Venketaraman	Glutathione is a tripeptide and antioxidant, and it synthesizes high levels of reactive oxygen and nitrogen intermediates used in regulating antigen-processing and ultimately intracellular mycobacterial growth.
53. Drug-resistant tuberculosis: past, present, future [53].	Chiang	Genetic resistance from random chromosomal mutation to anti-TB drugs is rare. Such resistance results in mycobacterial mutants gradually outnumbering susceptible bacilli and emerge as dominant. The reliability of drug susceptibility testing of second-line anti- TB drugs is questionable.
54. A Mycobacterium tuberculosis sigma factor network responds to cell-envelope damage by the promising anti-mycobacterial thioridazine [54].	Dutta	Thioridazine targets different pathways and can be used as multi-target inhibitor for M. TB.
55. Tuberculosis: Commentary on a reemergent killer [55].	Bloom	TB is the leading cause of death in the world from single infectious disease and there is little knowledge on pathogenesis and protection. Changes in social structure and public treatment programs are implicated.
56. Tuberculosis in patients with the acquired immunodeficiency syndrome. Clinical features, response to therapy, and survival [56].	Chaisson	Patients with TB and AIDS were more likely to be non-white and heterosexual IV drug users. It was found that 60% of AIDS group had at least 1 extrapulmonary site of disease compared to 28% in the non-AIDS group.
57. Relationship of the manifestations of tuberculosis to CD4 cell counts in patients with human immunodeficiency virus infection [57].	Jones	Extrapulmonary TB was found in 70% (30/43) of patients with CD4 counts less than or equal to 100, 50% (10/20) with counts 101–200, 44% (7/16) 201–300, and 28% (5/18) 300+ (p = 0.02). Acid-fast smears were more positive in patients with low CD4 counts. CD8 does not correlate with TB manifestations.
58. Sterilizing Activity of Pyrazinamide in Combination with First-Line Drugs in a C3HeB/FeJ Mouse Model of Tuberculosis [58].	Lanoix	The study found that while PZA is only beneficial in first 2 months in treatment of BALB/c mice, it is beneficial even beyond 2 months in C3HeB/FeJ mice
59. TGFbeta1-induced suppression of glutathione antioxidant defenses in hepatocytes: Caspase-dependent post-translational and caspase-independent transcriptional regulatory mechanisms [59].	Franklin	Their findings suggest that the suppression of GSH antioxidant defenses and the depletion of intracellular GSH may play role in enhancing TGFbeta-1 induced oxidative stress and potentiating apoptotic cell death
60. Circulating markers of free radical activity in patients with pulmonary tuberculosis [60].	CI	This study aims to measure circulating free radical markers in patients with TB. Their results showed markedly elevated levels of the three radical markers tested in all patients with TB.
61. Characterization of dendritic cell and regulatory T cell functions against M. tb infection [61].	Morris	It is hypothesized that GSH plays a vital role in enhancing the immune system. This study aims to investigate whether GSH plays a role in enhancing both the innate and adaptive immune system. The results found that GSH both inhibits M.Tb inside DCs and increases certain immune mediating molecules.
62. Recent advances in the diagnosis and treatment of multidrug-resistant tuberculosis [62].	Ahmad	This article discusses the advancement in treatment and diagnosis of multi-drug resistant tuberculosis, it also provides epidemiological information and statistics on Tb.
63. Controlled Clinical Trial Of Five Short-Course (4-Month) Chemotherapy Regimens In Pulmonary Tuberculosis [63].	The Lancet	This study was conducted on a group of 696 pulmonary tuberculosis patients who took antibiotic combinatiorifampin + isoniazid supplemented with ehtambutol or streptomycin followed with a treatment of chemotherapy. The results showed a 1% relapse in patients who received the chemotherapy treatment.
64. Comparative whole-genome sequence analysis of Mycobacterium tuberculosis isolated from tuberculous meningitis and pulmonary tuberculosis patients [64].	Faksri	This study focuses on isolated M.Tb sputum and CSF samples to seek genetic variants likely associated with underlying pathophysiology of the disease
65. Tuberculosis diagnostics: Challenges and opportunities [65].	Nema	This report discusses the limitations in diagnosis and treatments of tuberculosis meningitis.
66. Cerebral hemorrhage due to tuberculosis meningitis: A rare case report and literature review [66].	Zou	This case follows a report of a woman who presents with a walking instability, in trace reveal hemorrhage and leptomeningeal enhancement due to TB meningitis. Due to the patient’s complicated symptoms the article decides to report on the complexity and challenges of diagnosis TB. Meningitis using current methods.
67. PCR could be a method of choice for identification of both pulmonary and extra-pulmonary tuberculosis [67].	Amin	This Report focuses on the efficacy of PCR testing for M. Tb infections. It is found that PCR is a sensitive tool for the early diagnosis of MTb.
68. Polymerase chain reaction in the diagnosis of tuberculosis [68].	Jatana	This study evaluates the efficacy of PCR testing for detection of M.Tb infections. The study is conducted on hospitalized patients by examining a comparison of PCR body fluids. The study ultimately concludes that PCR may be a valuable tool for the diagnosis of CNS Tb.
69. Comparison of PCR with the routine procedure for diagnosis of tuberculosis in a population with high prevalence of tuberculosis and human immunodeficiency virus [69].	Kivihya-Ndugga	In low income countries ZN stain is the standard for diagnosis of TB due to its cheapness and simplicity. In this study AMPLICOR PCR System is tested in the diagnosis of Pulmonary TB. It was concluded that this system can be considered an alternative to the ZN stain and that sensitivity and specificity were increased and not affected regardless of HIV or Non-HIV status.
70. A breakthrough for the diagnosis of tuberculous pericarditis and pleuritis in less than 2 h [70].	Saeed	This study aims to evaluate GENEXPERT Assay and its validity for detection of M.TB. It was concluded that this assay is an innovative tool with high sensitivity and specificity, which may facilitate diagnosis and management of Tb Pleuritis and Pericarditis.
71. Lateral flow urine lipoarabinomannan assay for detecting active tuberculosis in people living with HIV [71].	Bjerrum	This study assesses the accuracy of the LF-LAM Assay to determine active Tb in HIV positive patients. Conclusions yielded results which state that LF-LAM has a sensitivity of 42% in the diagnosis of Tb on individuals who show symptoms.
72. Tuberculous meningitis: A uniform case definition for use in clinical research [72].	Marais	In an attempt to standardize criteria for the diagnosis and management of Tb meningitis, 41 international participants along with a consensus committee bound together to discuss treatment, management, pathogenesis and future of clinical research in the topic. It is the hope that by unifying the clinical diagnosis and management, decreased mortality and morbidity in Tb Meningitis should be observed.
73. Diagnostic standards and classification of tuberculosis in adults and children [73].	American Thoracic Society	This set of standards and classifications for Tb are installed in an attempt to provide a complete framework and understanding of clinical tuberculosis etiology and management.
74. Burden of tuberculosis at postmortem in inpatients at a tertiary referral center in sub-Saharan Africa: A prospective descriptive autopsy study [74].	Bates	Asymptomatic tuberculosis presents a clinical problem in that diagnosis is difficult due to the absence of symptoms and signs. This article reports on an autopsy performed in Lusaka Zambia in hopes of yielding information explaining the true burden of asymptomatic tuberculosis on affected patients.
75. The vitamin D-antimicrobial peptide pathway and its role in protection against infection [75].	Gombart	This article provides information on Vitamin D deficiency and its association with increased rates of infection. Specifically, the report provides insight on the possibility of using sunlight (environmental) and dietary vitamin D in the treatment of TB.
76. Real-Time Investigation of Tuberculosis Transmission: Developing the Respiratory Aerosol Sampling Chamber (RASC) [76].	Wood	TB is transmitted primarily through aerosol droplets. However, the adaptations which enable pathogens such as TB to avoid to exit the infected host, survive the external environment, adopt a form to enter the respiratory tract, and avoid innate defenses to establish nascent infection is unclear. A respiratory aerosol sampling chamber (RASC) was created to recreate the aerosol transmission.
